# An interview with Thomas Foutz, 2025 Epilepsia open prize winner for basic science research

**DOI:** 10.1002/epi4.70041

**Published:** 2025-05-19

**Authors:** Merab Kokaia, Piero Perucca

**Affiliations:** ^1^ Epilepsy Center, Faculty of Medicine Lund University Lund Sweden; ^2^ Department of Medicine (Austin Health) The University of Melbourne Melbourne Victoria Australia; ^3^ The Bladin‐Berkovic Comprehensive Epilepsy Program Austin Health Melbourne Victoria Australia

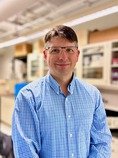

## TELL US ABOUT YOURSELF

I am an Assistant Professor in Pediatric Neurology at Washington University in St. Louis. My educational background includes a B.A. in Biophysics from Johns Hopkins University and an MD/PhD from Case Western Reserve University, where I specialized in Biomedical Engineering. My clinical training includes a pediatric neurology residency at Seattle Children's Hospital and an epilepsy fellowship at the University of North Carolina at Chapel Hill. I'm board‐certified in both Child Neurology and Epilepsy, with a clinical focus on treating children with refractory epilepsy. My research interests bridge computational neuroscience with animal models of epilepsy to develop novel neurostimulation approaches. I have published work on energy‐efficient neural stimulation, optical stimulation principles using optogenetics, and the spatial–temporal dynamics of neurostimulation in seizure models.

## HOW DID YOU BECOME INTERESTED IN CONDUCTING RESEARCH IN THIS FIELD?

My interest in neurostimulation research began during my graduate studies at Case Western Reserve University, where I worked under the mentorship of Dr. Cameron McIntyre at the Cleveland Clinic. I developed computational models to investigate neural responses to stimulation methods, including deep‐brain, optical, and peripheral nerve stimulation. This work culminated in novel approaches for energy‐efficient neural stimulation that could extend battery life and reduce the size of implanted pulse generators.

During my clinical training, I took care of children with treatment‐resistant epilepsy who continued to experience seizures despite multiple anti‐seizure medications. During my epilepsy fellowship, I received specialized training in brain stimulation therapies for refractory epilepsy. I appreciated that while many patients received some benefit from brain stimulation, too many continued to have persistent seizures. These clinical experiences showed me both the promise and limitations of current technology. This motivated me to bridge the gap between basic neuroscience research and clinical applications. I joined Washington University in St. Louis to receive additional training in animal models of epilepsy, physiological mechanisms of neurostimulation, and experimental trial design—to develop more effective, personalized neurostimulation therapies for drug‐resistant epilepsy.

## PLEASE EXPLAIN THE QUESTION YOUR STUDY ADDRESSED, AND HOW YOU DESIGNED IT

Our study investigated neurostimulation's optimal spatial and amplitude properties for inhibiting epileptiform activity in an acute hippocampal seizure model. The fundamental question was whether specific targeting of certain brain regions with optimized stimulation parameters could more effectively suppress seizure activity. I designed a high‐throughput series of experiments using the acute intrahippocampal kainic acid mouse model of status epilepticus. This model produces consistent and reproducible epileptiform activity, allowing systematic testing of multiple stimulation parameters. I stereotactically implanted six custom platinum‐iridium wire electrodes into six targets (bilateral CA1/dentate gyrus, ipsilateral subiculum, ipsilateral CA3, ventral hippocampal commissure, and medial septum).

After inducing seizure activity, I applied stimulation with varying amplitudes. I quantified epileptiform spike activity and evaluated spike frequency, amplitude, and variability changes. This approach allowed me to systematically assess location and amplitude combinations, providing a comprehensive map of how seizure activity responded to spatial and parametric variations in neurostimulation.

## WHAT WERE THE RESULTS AND HOW DO YOU INTERPRET YOUR FINDINGS?

Our results demonstrated that the spike‐suppressive effects of high‐frequency neurostimulation are highly dependent on both stimulation amplitude and location. Higher amplitude stimulation (250 μA) was significantly more effective than lower amplitudes. Epileptiform spiking activity was minimized after ipsilateral 250 μA stimulation of the CA1 and CA3 hippocampal regions, with spiking reductions of 21.5% and 22.2%, respectively.

Interestingly, we also discovered regional differences in response patterns. CA1 stimulation produced broader effects, suppressing activity in multiple regions, including the ipsilateral subiculum, CA1, and CA3. In contrast, CA3 stimulation effects were more focally contained to the stimulation site. We also found that stimulation at the ventral hippocampal commissure increased spiking frequency and amplitude, suggesting improper targeting could worsen seizure activity. The spike‐suppressive effect showed a distance‐dependent relationship at higher stimulation amplitudes, with more potent effects closer to the stimulation source. This effect was most pronounced with ipsilateral CA3 stimulation. Additionally, we observed that the suppressive effects were time‐limited, with activity gradually returning to baseline, indicating the need for repeated or sustained stimulation for lasting effects.

These findings suggest that precise targeting of stimulation to specific epileptogenic regions (particularly CA1 and CA3 in temporal lobe epilepsy) with appropriately high stimulation amplitude could significantly improve the efficacy of neurostimulation therapy. The results help explain why clinical approaches with limited spatial coverage and empirical parameter selection often produce suboptimal outcomes. These results provide some insight toward more rational, targeted neurostimulation protocols.

## WHAT ARE THE NEXT STEPS THAT YOU PLAN TO TAKE, AND WHAT ARE YOUR CAREER GOALS?

I plan to extend my work to chronic epilepsy models that better reflect spontaneous seizures in humans, focusing on developing closed‐loop stimulation systems with optimized parameters. I'm also investigating thalamic targets while expanding stimulation parameters to include additional frequencies, pulse widths, and waveforms. Using computational modeling with in vivo recordings, I aim to understand neurostimulation's mechanisms for seizure suppression. I aim to establish an independent research program to translate laboratory discoveries into effective therapies for drug‐resistant epilepsy.

## WHAT DOES THE EPILEPSIA OPEN PRIZE MEAN FOR YOU, YOUR LABORATORY, RESEARCH INSTITUTE, AND YOUR FUTURE?

The Epilepsia Open Prize validates our mission to improve neurostimulation therapy for epilepsy. This prize will help accelerate our transition to chronic epilepsy models and the development of adaptive closed‐loop stimulation systems. This recognition supports our mission to transform novel brain stimulation therapies into targeted treatments that significantly improve the quality of life of people with refractory epilepsy.

Read the winning article: Spatial and amplitude dynamics of neurostimulation: Insights from the acute intrahippocampal kainate seizure mouse model

## CONFLICT OF INTEREST STATEMENT

The authors report no competing or financial interests.

## Data Availability

Data sharing is not applicable to this article as no new data were created or analyzed in this study.

